# Performance analysis of network automation techniques for dense IP networks

**DOI:** 10.1038/s41598-026-40975-9

**Published:** 2026-03-20

**Authors:** Mohammad M. Abdellatif, Osama Desouki, Mohamed AbdelRaheem

**Affiliations:** 1 Electrical Engineering Department, The British University in Egypt, Cairo, 11837 Egypt; 2 Electrical Engineering Department, Faculty of Engineering, Assiut University, Assiut, 71111 Egypt

**Keywords:** Engineering, Mathematics and computing

## Abstract

Network automation is an emerging technology which gained a lot of traction over the past few years. NA can be used at multiple levels, such as topology creation, configuration generation, and testing. It can save a great amount of time and effort compared to conventional ways. Here, the automated topology creation tools EVE-NG, Pllama, and Container LAB are evaluated. For configuration generation, Nokia’s Komodo was tested and compared with an automated configuration using python and manually using an Excel sheet. Finally, various test cases were executed in CLASSIC-CLI, MD-CLI, and NETCONF to evaluate the automation performance on the testing level. The results showed that automation greatly reduces the time spent in all the stages of the network deployment mentioned above. In addition, automated topology creation is shown to be 4.5 times faster than in the manual case, configuration generation is about 10% better than the manual case, and test execution time is 11 times faster than manual testing.

## Introduction

Network Automation (NA) is a concept that is widely used in the telecommunications industry today due to the wide expansion in the number of nodes used in different projects such as 4G-5G migration and data centers^1,2^. Furthermore, the benefits of automation can be of great use to businesses, reducing operational expenses (OPEX), time, and human errors; therefore, contributing to companies’ growth and stability^3^. According to Nokia and Cisco, NA is defined as performing any repetitive task that is supposed to be performed manually, automatically. The automation process releases ordinary tasks such as performance monitoring, configuration, testing, and upgrading from network administrators, thus allowing for innovation and improving overall performance. In IP networks, automation can be used in different phases of the project life cycle, from planning and deployment, to testing and monitoring^4^. NA increases the efficiency of the network and its ability to respond to vigorous dynamic changes in today’s demanding network usage flow. This can be usually done by programmatic features such as using Python-based scripts and templates^1,3^.

Over the past few years, vendors have started to develop software capabilities and tools that help in the automation process^2^. Some of these tools are open source like python, Robot Framework, and Jinja templates used in testing and configuration deployment. While others are proprietary, as Nokia Services Platform (NSP), and Cisco Network Services Orchestrator (NSO) used for Network Orchestration^2^. Network orchestration refers to the automated management and coordination of network elements and resources to enable efficient and reliable operation of network services. Additionally, Mobile Service Providers (MSPs) deploying 5G networks encounter a problem in the number of IP nodes to be configured, tested, and monitored^5^. Human manual intervention in a large network of thousands of nodes is time-consuming and prone to errors. Therefore, a standard automation method in configuration generation and testing is required to avoid such errors^2,5,6^. The concepts of automation and standardization complete each other and allow for better performance, as it simplifies troubleshooting^7^. This research aims to analyze the performance of NA techniques on multiple stages in the network’s deployment, mainly in routers’ configuration generation and testing. For configuration generation, we use Jinja Templates in combination with an Excel sheet to generate the Jinja variables, thus facilitating the process of configuration generation and minimizing configuration errors. For testing, we compare different tests done on classic Command Line Interface (CLI), Model Driven Command Line Interface (MD-CLI), and Network Configuration Protocol NETCONF. Furthermore, an auto config-generation tool “Komodo 2” is used to create a virtual lab with Nokia 7750 SR OS routers on a container Lab (CLAB) to save time on topology creation. Komodo is a configuration generation tool that is proprietary to Nokia currently in its beta phase of development. The Jinja templates we create are to be used in case of missing templates for services and protocols in the Komodo tool.

To validate the work, various automated tests are executed on the system to verify its functionality. Test cases based on classic CLI are developed in Model-Driven Command Line Interface (MD-CLI) and NETCONF using Robot Framework and SR OS Automation Testing Service (SATS) are performed and compared. The execution time is the main metric is tested on the 3 systems.

A virtual Lab is created on a virtual emulator EVE-NG to place the configuration files of the auto-configured devices. This is performed to compare the execution time of manual topology creation with the automated topology creation that is done in other tools. Some Multi-Protocol Label Switching (MPLS) test cases are developed along with some system Test Cases measuring temperature, CPU usage, and link utilization.

The contribution of this work can be summarized as follows:

- End-to-end automation evaluation: This paper provides the first end-to-end performance evaluation covering all three stages of network automation - topology deployment, configuration generation, and automated network testing - in one unified framework. Prior studies usually examined only one or two of these stages in isolation. In contrast, we compare all three stages together, offering a comprehensive view of the automation pipeline.
- Broad Toolset Comparison: We compare multiple tools side-by-side at each stage of automation:
  - Topology emulation: EVE-NG, Containerlab, and pLlama.
  - Configuration generation: Nokia’s proprietary Komodo tool, our open-source Python scripting approach, and traditional manual methods.
  - Network testing: Classic CLI, MD-CLI, and NETCONF interfaces (automated via Robot Framework).

Conducting a broad comparison under identical conditions is novel, since earlier work often focused on a single vendor’s tools or a single aspect of automation. Our study evaluates a wider range of tools and methods in a consistent environment.

- Custom automation enhancement: We developed a custom Python-based YAML generation script for Containerlab and show that it dramatically speeds up network deployment - reducing deployment time from about 9 minutes to  2 minutes (a  4.5$\times$ speed-up). This practical improvement has not been reported in prior literature and demonstrates how a small tooling enhancement can yield significant efficiency gains in network operations.
- New performance data for MD-CLI vs. NETCONF: To our knowledge, we present the first published performance data comparing Nokia SR OS’s Model-Driven CLI (MD-CLI) and NETCONF interfaces for automated network testing on a realistic IP/MPLS network. In our results, NETCONF executes test cases roughly 10$\times$ faster than MD-CLI (and about 11$\times$ faster than the classic CLI) on average. This highlights the major benefits of model-driven management (NETCONF/YANG) for speed and efficiency in network automation.
- Operational trade-off analysis: Beyond raw performance metrics, we discuss important operational trade-offs in adopting automation. For example, we consider network engineers’ familiarity with classic CLI versus the learning curve of MD-CLI/NETCONF, and we weigh proprietary solutions against open-source alternatives.

The rest of the paper is organized as follows. “Background and literature review” gives a summary of previous work performed on the topic in literature. “Experimental work and main results” lists the experimental setup and the main results. “Analysis” presents an analysis of the results. And finally, “Conclusions and future work” concludes the paper.

## Background and literature review

In the past, the bottleneck to achieving high data throughput was the communication media. However, As the backbone links are converting to fibre optics, the bottle neck becomes the routers rather than the links. This has created a need for parallel development leading to increasing processing power and memory for routers to meet the forwarding demands and manage protocols rapidly.

Inside any router, the control plane organizes routing entries and selects routes, while the data plane forwards packets. These planes have different needs and can be separated^8^. This separation was the idea behind Software Defined Networks (SDNs) which define a method to move from legacy networks to more intelligent programmable networks with a controller or orchestrator to control the forwarding options defining the control plane, and a data plane in which network nodes perform packet forwarding^9^.

### Software defined networking (SDN)

**Fig. 1:**
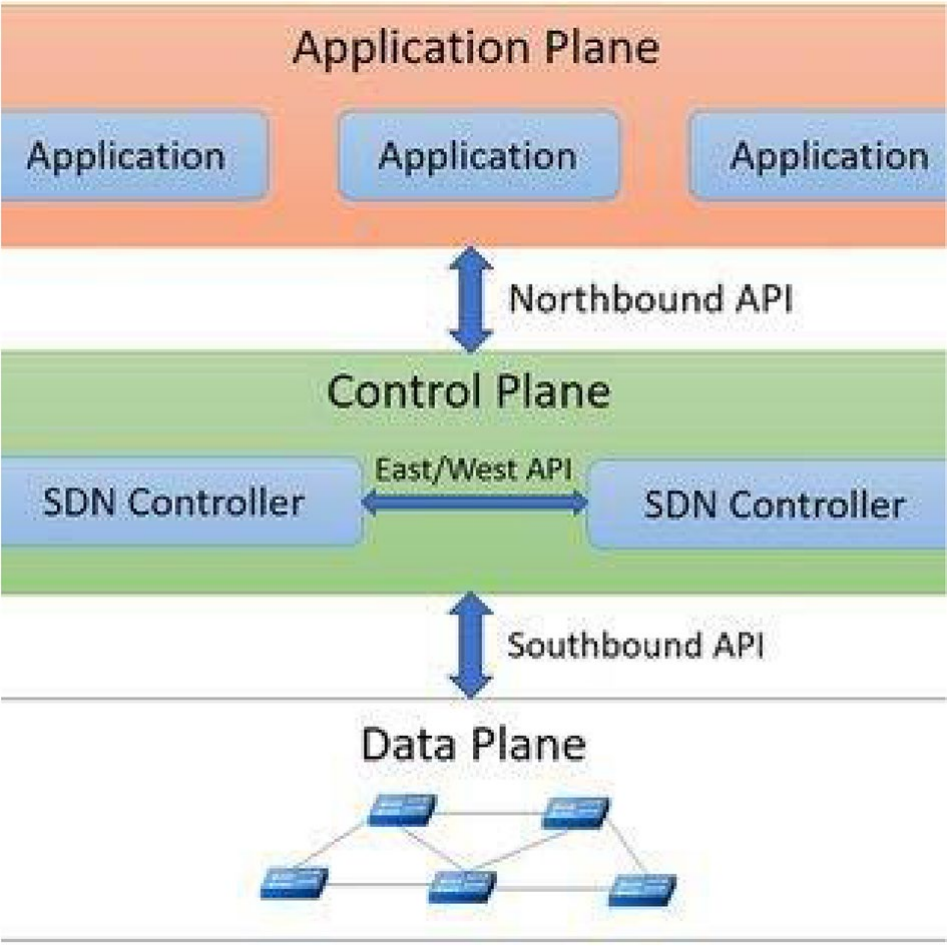
SDN architecture^9^.

The SDN reference model is a three-layer stacking model: infrastructure, control, and application layers as shown in Fig. 1. The infrastructure layer includes networking devices like routers and switches, responsible for data collection and processing packets based on control layer rules, representing the data plane. The control layer connects infrastructure and application layers, managing the infrastructure through rule-based architecture and interfaces. The application layer meets user requirements with applications such as routing and monitoring, enhancing performance through centralized real-time control. Application logic is translated by the control layer and implemented in the infrastructure layer as actionable rules^7^.

The idea of SDN was proposed in a set of projects^7^ but none has the complete view of the SDN in terms of maturity and scalability. Firstly, some projects deployed active networking concepts, where software is used to manage the network in a real-time fashion. Moreover, modifying routing software in PC Hardware-based routers was made possible by software like eXtensible Open Router Platform XORP which supports routing protocols like OSPF, BGP, RIP, and PIM^10^. Other systems like BIRD and Quagga deploy a dynamic IP Routing solution on Linux and Unix machines^10^. Secondly, Separating the control plane from the data plane is not a novel topic since some projects have deployed the idea of decoupling since the early 2000s as Routing Control Platform (RCP) in which a centralized routing control is applied to BGP path computation^11^. Third, Internet Engineering Task Force (IETF) introduced Forwarding and Control Element Separation (ForCES); a framework for the communication between the decoupled planes defining signaling and protocols required between control and data plane^12^. Moreover, Path Computation Element (PCE, defined in RFC 4655) was introduced to overcome the limitations of Constraint-based path computation (CSPF) in which Label Switched Paths (LSP) are computed separately via a controller or orchestrator -such as Nokia NSP or Cisco NSO- in an MPLS network^13^.

### Network configuration protocol (NETCONF)

In multi-vendor ISP environments, traditional CLI-based configuration and fault management face challenges due to diverse command structures. Simple Network Management Protocol (SNMP), introduced in 1988, became widely adopted but lacked robust configuration capabilities. The IETF addressed this with the standardized Network Configuration Protocol (NETCONF) in 2006, aiming to standardize network configuration management. NETCONF offers several powerful features for network automation:

- Datastore separation: It distinguishes between Configure and State datastores, where “Configure” contains configuration commands and “State” holds operational data. This standardizes configuration across vendors by defining syntax and semantics.
- Remote procedure call (RPC) support: NETCONF enables servers to execute procedures on remote clients like local execution, facilitating efficient management tasks.
- Legacy SNMP interoperability: It interoperates with SNMP via mediators, translating XML to MIBs for legacy SNMP devices, ensuring backward compatibility.
- Transactional configuration: Supports atomic transactions by storing changes in a candidate configuration datastore. Changes are activated or discarded as a whole, ensuring consistency and reliability in network configurations.

In this work, NETCONF is used to test and compare the different automation techniques used.

### Robot framework

Network Test Automation (NTA) plays a crucial role in verifying system operations and functionality within project life cycles, aiming to reduce time and human errors, which makes automating network tests essential as manual testing can be repetitive and error-prone, leading to inaccuracies. Robot Framework, a widely used tool in automation testing, simplifies scripting with its human-readable syntax and supports integration with various systems and devices. It allows importing libraries like Secure Shell (SSH), Selenium for web testing, and Representational State Transfer (REST) to enhance testing capabilities. The framework’s structure includes settings for importing libraries and defining test suites, variables for global definitions, test cases for defining criteria and automation steps, and keywords for reusable functions across test cases, promoting clarity and efficiency in test automation.

### Literature review

Many research papers have tackled the issue of network automation in the past, in this subsection, we list some of the most prominent ones. Altalibe et al.^14^ provided a Python-based automation solution for traditional IP networks. They developed and tested what they referred to as Automation Scripts Code (ASC) to configure Cisco devices, achieving fast configuration times across multiple devices. The paper focuses on practical, vendor-specific automation with performance optimization, but applicability to non-Cisco or multi-vendor environments is unclear. Our work applies to both proprietary as well as open-source environments.

Yuansa et al.^15^ provided a comparison of libraries for automating OSPF and BGP configuration in traditional IP networks. They used Paramiko and Telnetlib to automate IP address assignment and routing protocol configuration, focusing on performance metrics like delivery time. The paper directly addresses automating routing protocols (OSPF, BGP) in legacy distributed environments; however, it does not rely on SDN/NFV concepts. Our work uses SDN extensively for the automation.

The authors in^16^ proposed an implementation of network automation for BGP in traditional IP environments. Their work used Ansible to automate BGP configuration, IP assignments, and backups in a live, non-virtualized network via SSH. Like our proposed work, the paper focused on practical scripting and automation for distributed routers, aligning well with traditional IP network automation goals.

In^17^, Bringhenti et al. studied automation in firewall configuration focusing on packet filters which are considered one of the most common firewall technologies used in computer networks. The authors proposed an automatic method to define the allocation scheme and configuration of packet filters in a virtual network. Moreover, they have evaluated their proposed methodology using multiple metrics and tests on both synthetic and real use cases and compared it to state-of-the-art solutions. In this work, we perform a similar task in network automation in general, but we have not used packet filters.

While much of the current literature focuses on specific network protocols, the scope of automation is expanding rapidly into industrial sectors. Recent studies on industrial automation, such as^18^, discuss ’Production plant and warehouse automation with IoT and Industry 5.0,’ highlighting frameworks that integrate intelligent systems similar to the network automation techniques proposed here. Integrating these broader industrial perspectives helps contextualize the practical relevance of our proposed approach beyond telecommunications.

After reviewing the existing literature on network automation, we observed that many prior works address individual parts of the automation process but not the entire pipeline. Table 1 below provides a summary of representative studies from the literature, highlighting the tools/techniques they used, their evaluation focus, and their limitations. This comparison clearly shows the gaps that our work aims to fill.

**Table 1:** Summary of related work in network automation.

References	Tools/techniques used	Scope of evaluation	Key limitations/gaps
Mazin et al.^1^	Python scripting for device configs	Automated network device provisioning- measured configuration speed on Cisco routers	Focused only on configuration stage; single-vendor (Cisco) environment; did not evaluate topology or testing automation
Altalebi et al.^14^	Custom automation scripts on large networks	Optimization of deployment time in a large IP network—introduced scripts to speed up configuration	Evaluated automation in one domain (mainly config); specific to Nokia routers; no coverage of automated testing procedures
Alfaresa et al.^15^	Network automation library (Python-based)	Automated routing protocol configuration (IGP/BGP)—compared manual vs. automated	Focused only on configuration of routing protocols; did not address topology setup or network testing; limited to configuration accuracy and time
Harahus et al.^19^	Network lab platforms (EVE-NG vs. GNS3 vs. VIRL)	Comparison of network emulation tools—evaluated setup complexity and performance of different lab environments	Examined lab tool capabilities and performance, but not the automation of configuration or testing; no end-to-end automation analysis
Fawzy et al.^20^	DevOps pipeline concepts for networking	Conceptual framework for network automation using DevOps approaches—discussed integration of CI/CD for network configs	High-level and theoretical (DevOps approach); provided no performance metrics; did not implement or measure actual automation tool performance

Table 1 shows that previous studies typically address specific aspects of automation. In contrast, our work covers the full lifecycle (lab setup, config generation, and testing) and evaluates multiple tools in each category under the same conditions. For example, unlike Mazin et al. or Alfaresa et al., who focused solely on speeding up device configuration on one vendor’s equipment, we evaluate an end-to-end scenario across multiple vendors’ tools and multiple stages. And while works like Harahus et al. compare network lab environments, they do not tackle the automation of configurations or tests, which is a gap our study fills. By synthesizing insights from these prior efforts and addressing their limitations, our research is positioned as a broader and more integrated evaluation of network automation techniques.

## Experimental work and main results

### Experimental setup and environment

All experiments were conducted on a dedicated server to ensure consistent results. The server is a Dell PowerEdge R740 with dual 8-core Intel Xeon Silver 4210 CPUs (16 cores total at 2.20 GHz) and 64GB of RAM, equipped with a 1TB NVMe SSD for storage. The host operating system is Ubuntu 20.04 LTS (Linux kernel 5.4). We deployed the network lab tools on this server as follows:

- EVE-NG: Run as a virtual machine (Community Edition v2.0.3) on a VMware ESXi 7.0 hypervisor on the server. The VM was allocated 8 vCPUs and 16GB of RAM.
- Containerlab (CLAB): Run natively on the Ubuntu host (using Docker Engine 24.0). Containerlab version 0.41 was used. (Containers were allowed to use the host’s CPU cores with default scheduling; memory usage for CLAB peaked around  12GB during the largest topology deployment.)
- pLlama: Run as a local application on the same host. pLlama was a similarly lightweight tool and used system resources comparable to Containerlab.

For network device instances, we used Nokia’s Virtual Service Router (VSR) software (SR OS v21.10) for all emulated routers across all platforms (EVE-NG and Containerlab). This means every network node in our tests was running the same network OS image and version, ensuring a fair comparison between EVE-NG and CLAB deployments. By using identical router images and configurations, we eliminate inconsistencies—the differences observed are purely due to the automation tool or method, not the network elements themselves. To ensure fairness and reproducibility, we kept the test environment controlled and consistent for every trial. The server was dedicated to these experiments with no other significant workloads running. During all measurements, we monitored the host’s CPU utilization and network usage to verify that no background processes or external traffic interfered. The lab network was isolated (no Internet or enterprise traffic) so that automation tasks were the only load on the system. In summary, each tool was evaluated on a level playing field - using the same hardware resources, identical network scenarios, and no external load - so that performance comparisons between tools are valid.

### Topology deployment

**Fig. 2:**
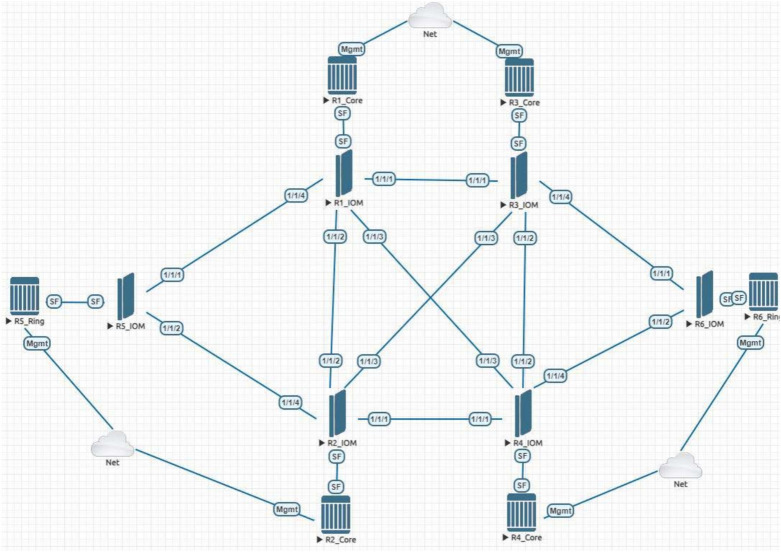
EVE-NG topology of 6 routers for IP/MPLS.

**Fig. 3:**
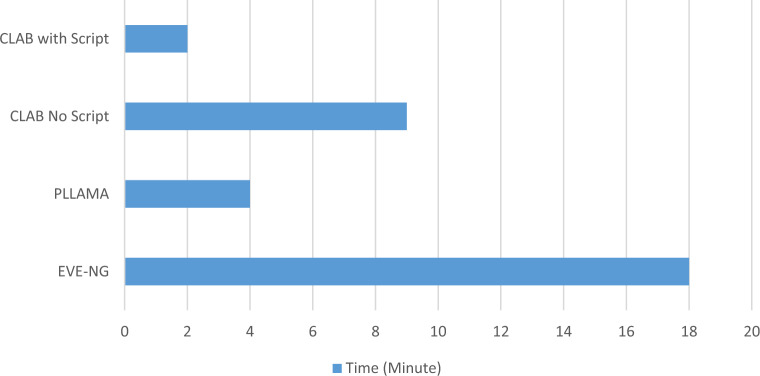
Comparison of time in minutes taken to create a topology on different tools.

Three topology design software tools—EVE-NG, Pllama, and Container Lab (CLAB)—are deployed and tested. These tools require pre-configuration tasks such as setting up the server for emulated images and configuring the server quota based on design requirements. Tests have been conducted to deploy the 6 routers topology shown in Fig. 2 on the three mentioned tools with time in minutes shown in Fig. 3.

We chose a 6-node IP/MPLS core network topology for all experiments. This moderate-sized topology represents a simplified core network that is large enough to be realistic but small enough to manage easily. The reason for using six nodes is to focus on automation overhead rather than complex network behavior. In a much larger topology, we would expect deployment times and configuration efforts to grow roughly linearly with the number of nodes, without fundamentally changing the relative performance of the tools. Thus, the 6-node scenario provides clear results for comparison. Moreover, in Section 5, we note that future work will explore larger-scale networks to confirm that our conclusions hold at scale.

CLAB uses a Yet Another Markup Language (YAML) file to deploy the configuration. Normally the YAML file is populated manually by the administrator. In this work, we added an extra step where an excel sheet is integrated with a python script that fills the variables for the YAML file such as nodes and connections. This technique is referred to as “CLAB with script” in Fig. 3. This modification reduced the time required to complete the YAML file by 4.5 times compared to the traditional method.

### Configuration generation

Configuration Generation is deployed with three methods: manually generating configuration files, with a python script developed for this project that takes its input from an excel sheet and generates YAML files for variables that will be used to generate the configuration from jinja templates, and finally, using Komodo tool that is proprietary to Nokia.

**Fig. 4:**
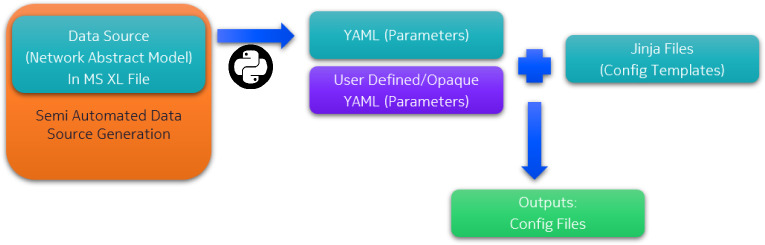
The YAML file is generated automatically from an excel sheet that is filled by the user.

- Manual configuration Two network administrators were tasked with the creation of the topology shown in Fig. 3 and then to configure a Virtual Private LAN Service (VPLS) between router R5 and R6 passing through the Four core routers R1–R4. For the topology creation, the two administrators took about 18 Minutes to complete the task. As for the VPLS configuration, the first administrator took 133 minutes to complete the task, while the second administrator took 67 minutes to complete it. Four errors resulted from the manual configuration: two for each administrator.
- Komodo Komodo is a configuration generation tool that is proprietary to Nokia. Komodo integrates many toolsets that can be used to generate configuration, provide a physical view of the topology, deploy labs, and run automated test cases on the network. Komodo uses an excel sheet and jinja templates to generate configuration files for nodes and to generate the YAML file for the CLAB to be deployed later. Additionally, Komodo can detect design flaws while generating the outputs. However, as Komodo is a proprietary technology, it cannot be used to configure devices by other vendors. And integrating it with open-source solutions is challenging. And so, it is only discussed here as a benchmark for the testing of the proposed solution.
- Proposed python script Like Komodo, the proposed python script uses a user generated Excel sheet, as shown in Fig. 4, to take configuration input from the administrator. Thus, designate a human readable interface and convert this input to a YAML file. The YAML file is then integrated into a jinja template to create the required configuration file for CLAB.

To test their performance, the automated solutions have been tasked with creating the same configuration as the manual version. Both were time stamped, and their errors were evaluation after finishing. Both Komodo and the proposed solution outperformed the manual configuration by taking less than 10% of the total time required for the manual network configuration with zero errors.

### Testing

Network Testing is an essential step in any project’s life cycle to ensure that the network meets the design requirements and operates smoothly. Normally, test cases are applied to the network to test different aspects of the network, such as performance, reliability, and security. Additionally, the evaluation of the network can be done by measuring metrics such as throughput, delay, and jitter. By measuring such values, network administrators can determine the ability of the network to run specific applications and services without significant delays or interruptions, especially for real-time traffic such as online shopping, gaming, and finance.

Network testing can be accomplished by manual testing, where a command line interface is used to collect information from a node and analyze it by a network administrator. Or by automated testing, through programming tools such as Robot Framework and Python. Automated network testing is an emerging technological trend in modern network management and development, leveraging testing quality and performance. It increases accuracy and documentation quality (e.g. highlights in reports). Additionally, it reduces testing time significantly to suit short maintenance windows, thus, reducing operating expense (OPEX).

#### Manual testing

To evaluate the network using manual testing, a test scenario is applied. In this work, the test scenario selected is the verification that ICMP Unreachable messages can be disabled or enabled on L3 interface on SR OS device. Receiving an ICMP Unreachable message usually means that a frame was discarded because it could not reach its ultimate destination. Disabling ICMP Unreachable messages can prevent possible DDoS attacks. The preconditions for this test are:

- Two devices connected to each other with L3 interface.
- An unreachable dummy route which is predetermined before the test to create ICMP Unreachable messages.
- ICMP Unreachable messages should be seen in debug.

This Manual test case that follows the above point with documentation took about 45 minutes. It is estimated that without documentation it would take 15 minutes. This shows while manual testing can be more accurate in network evaluation, it is time and resource use is heavy. In the next subsection, we evaluate the performance of automated testing tools such as: Classic CLI, Model-Driven CLI and NETCONF for the same test scenario.

#### Automated testing

SATS is a Robot Framework Library designed to automate operations on Nokia’s SR OS. SATS provides keywords for common router tasks like connecting to nodes, executing commands, reading outputs, and retrieving configuration or state data. Access methods include classic CLI, MD-CLI via SSH, and NETCONF, each with its own architecture and keywords. In this work, these methods are tested on Nokia 7750 SR OS, with comparisons based on complexity and time.

Executing the test case in Classic CLI using parsing templates for the returned outputs, returns a structured list or list of dictionaries where looping them is made easier. The same test case used in the manual testing above was executed three times and took an average of 18.376 s to complete.

Classic CLI was designed for human machine interaction, where it lacks a standard method for naming conventions and is vendor specific. The output of classic CLI contains tables and strings in most scenarios and does not have a structured syntax or parsing template. Parsing templates are tailored to every command output per vendor to allow machines to interact with it. This is a completely manual test.

Model Driven CLI (MD-CLI) overcomes the mentioned limitations in classic CLI where MD-CLI makes use of YANG data models. The output from MD-CLI is in JSON format making it easy for machines to understand. The same test case used in Classic CLI is used but in model-drive format. The scenario is tested three times and took an average of 15.418 s. The SSH connection took 4.3 s on average, while the test step took 8.3 s on average. This is a Hybrid test.

NETCONF is a network management protocol designed for consistent and automated network management. It supports operations to edit, delete, and merge configurations on network devices. NETCONF uses YANG data models and supports transactional configurations with three datastores: running, candidate, and startup. Its key advantages include standardization, allowing management of different vendor devices with a uniform approach, and facilitation of automation, enhancing efficiency and reducing human errors. This is a completely automated test.

Like MD-CLI, NETCONF doesn’t require parsing outputs. The outputs are retrieved from the networking device in an XML format. Get State and Get Config operations are used to collect state and configure datastores, respectively. Filtering the received output from the router to get the required piece of data should be made carefully using xml filters. The three trials took on average 3.219 s to execute.

Additionally, to evaluate the manual testing methods, a combined test was carried out for four test cases, namely, The Target LDP Session, Available Memory Alert, Displaying Message of The Day (MOTD), and Defining User Profiles. The combined test was executed for three automated testing methods, cli, md-cli, and NETCONF. All the test cases have been executed on the 6-router topology shown in Fig. 2 above. The results are available in Table 2 with time in seconds.

**Table 2:** Execution time (in seconds) for a collection of four test cases in classic-CLI, MD-CLI, and NETCONF.

Test	Classic-CLI (manual)	MD-CLI (hybrid)	NETCONF (automated)
Target LDP session	19	19	3
Available memory alert	228	171	20
MOTD	6	12	1
Defining user profiles	56	87	4
Total time	308	289	28

## Analysis

In this section, we analyze the results obtained in the previous section.

Throughout the aforementioned experiments, we collected relevant performance metrics (primarily time taken, and error counts where applicable) for each tool or method. Each experiment was repeated 5 times to account for run-to-run variability. Unless otherwise noted, the results we report are the average of five runs. We also computed the standard deviation for key metrics to understand the variability in performance. In the results sections, we include these variance measures. This way, we can assess not only the average performance of each approach but also the consistency of that performance across multiple trials. By following this procedure, we ensured that our comparisons are rigorous and statistically sound. The consistent environment and multiple trial runs allow us to confidently highlight differences between automation tools. In the next section, we present the results for each stage of the automation process, along with analysis and discussion of the findings.

### Topology deployment

Using our testbed, we measured how quickly each tool could deploy the 6-node network topology. The results show clear differences in deployment speed:

- EVE-NG required about 540 s (± 15 s) on average to launch and configure the six virtual routers. This was our baseline, as EVE-NG orchestrates virtual machines for each router which takes some time.
- Containerlab, using our automation enhancements, was dramatically faster. With the Python-generated YAML topology file, Containerlab deployed the same network in roughly 130 s (±8 s) on average. This is about a 4$\times$–5$\times$ speed improvement over EVE-NG’s deployment time. In other words, what took nine minutes with traditional methods was done in just over two minutes with Containerlab. The improvement is largely because Containerlab leverages lightweight Docker containers and our automation script optimizes the setup of those containers.
- pLlama had deployment times in between the above two. On average, pLlama’s deployment time was closer to EVE-NG’s. (For instance, pLlama was moderately faster than EVE-NG, but not nearly as fast as Containerlab.) The difference between pLlama and EVE-NG was smaller, indicating comparable performance in launching the network, whereas Containerlab clearly outpaced both.

These results highlight the benefit of using container-based virtualization for rapid lab setups. In particular, our custom YAML automation script contributed substantially to Containerlab’s efficiency. We found that without this script, setting up the Containerlab topology could take around 8–9 min (similar to EVE-NG’s time). By automating the topology definition and configuration with our script, the Containerlab deployment time dropped to  2 min. This showcases how a small tooling enhancement can eliminate repetitive manual setup steps and dramatically speed up network instantiation.

### Configuration generation

In this stage, we compare how long it takes—and how error-prone it is—to generate device configurations using three approaches: manual editing, Nokia’s Komodo tool, and our automated Python script. We also clarify what we mean by the configurations being “better” or “worse” by considering multiple factors: time taken, errors encountered, and scalability.

Time performance: To establish a strong baseline, for a network of six routers, we found that doing the configurations manually (writing configs by hand, even with the help of an Excel template) took on the order of 45–50 min to complete all devices. This baseline allows us to quantify the performance of the State-of-the-Art (SOTA) Komodo tool and our proposed Python script. Using Nokia’s Komodo tool was a bit faster—roughly 45 min for the whole network—since Komodo automates many steps but still requires some user interaction and was limited to Nokia-specific templates. Our Python script was the fastest method, finishing the configuration generation in around 40 min for all six nodes. In other words, the automated Python-based approach was about 10–15% faster than the purely manual process for this scenario. This is what we originally meant by saying automation was “about 10% better” in terms of speed: the scripting approach saved several minutes out of  45 min, which is a noticeable improvement. Komodo’s time was in between, showing a benefit over fully manual work but not as fast as our custom tool.

Error rate: Speed is only one aspect of “better” - reliability is another. In the manual configuration trials, we typically encountered a couple of minor syntax errors (on average 1–2 errors per full configuration run). These were things like a mistyped command or an inconsistent parameter, which had to be found and corrected by the engineer, adding to the effective deployment time and effort. Such human errors are not surprising—it’s well known that manual network configuration is error-prone, and indeed many network outages stem from manual config mistakes. Nokia’s Komodo tool, being an automated generator, produced no syntax errors in the configurations it supported—it has built-in checks that prevented malformed commands. However, Komodo could not generate every aspect of our desired config (since it’s limited to certain templates and was in beta), so a few manual tweaks were needed for unsupported features, which we don’t count as “errors” but do require additional effort. Our Python script produced zero configuration errors in all runs. Once the templates were written correctly, the script reliably output error-free configs for every router. This demonstrated one big advantage of automation: removing human error from the configuration process. The Python approach essentially eliminated typos and inconsistencies, resulting in configurations that worked on the first try for all devices.

Scalability: We also considered how each approach would scale if we had to configure a larger network or repeat the process frequently. The manual method obviously does not scale well—adding more devices increases the work linearly (or worse, if human fatigue leads to more errors on a bigger network). A manual approach might be feasible for half a dozen routers, but would be extremely time-consuming and error-prone for, say, 50 or 100 routers. Nokia’s Komodo can handle more devices relatively easily (as it’s an automated tool), so in terms of scaling to many nodes, it’s much better than manual. However, Komodo is a proprietary Nokia tool—it only works for Nokia SR OS devices and requires a license—which limits its use in multi-vendor networks and for researchers or engineers who don’t have access to Nokia’s ecosystem. Our Python-based tool, on the other hand, is designed with scalability and flexibility in mind. Generating configs for additional routers simply means adding more entries to the input Excel sheet; the script can loop through dozens of devices. The runtime grows roughly linearly with the number of devices (in our tests, it took only about 8 s per additional router to render the config via our templates). This is very efficient compared to the minutes of work each additional router would impose in a manual workflow. Moreover, because our solution is based on templates and standard libraries, it can be adapted to any vendor - by switching out the Jinja2 templates, the same script can generate configurations for Cisco IOS, Junos, or other platforms. This means our approach can scale not just in size but across different network environments, avoiding vendor lock-in.

To summarize these findings, we created Table 3 to compare the three configuration methods side by side:

**Table 3:** Manual vs. Komodo vs. Python—configuration generation comparison.

Approach	Avg. time (6 routers)	Errors encountered	Scalability and notes
Manual (Excel-aided)	$\tilde{50}$ minutes	1–2 errors on average	Effort grows linearly with network size; prone to human errors as the number of devices increases
Komodo (Nokia tool)	$\tilde{45}$ minutes	0 (automated checks)	Can handle more devices (automation helps) but Nokia-only (proprietary tool, requires license); limited support for non-Nokia gear
Python Script (ours)	$\tilde{40}$ minutes	0 errors	Scales easily—adding devices only adds a few seconds each; templates can be modified for any vendor (multi-vendor capable, open-source)

### Testing

We designed a set of automated test cases to verify various aspects of network operation, and we ran them using the three different interfaces (Classic CLI, MD-CLI, and NETCONF). The tests we included cover a spread of typical network behaviors and failure detection mechanisms:

- ICMP unreachable: We test whether routers generate ICMP “Destination Unreachable” messages when they cannot forward a packet. This is a fundamental IP networking behavior that has implications for security (e.g., some networks disable these messages to avoid revealing information or to mitigate certain types of DoS attacks). Verifying ICMP Unreachables ensures our routers are configured correctly to handle undeliverable traffic, which is a common real-world check (to prevent blackholing traffic or to ensure security policies are enforced).
- LDP session establishment: In an MPLS network, Label Distribution Protocol sessions between adjacent routers are crucial for setting up label-switched paths. Our test checks that an LDP session comes up successfully between two routers. This is representative of a core control-plane health check for MPLS networks—if LDP (or any routing protocol session) fails, it indicates an issue that could impact services.
- MOTD banner display: We retrieve the “Message of the Day” banner from the routers. While this is a simple configuration item (it doesn’t affect traffic), it serves as a quick test of configuration consistency and remote access. Ensuring the MOTD is correctly set and can be read is like a proxy for verifying that configurations were applied properly and that the device’s state can be queried. It might catch issues where parts of the config didn’t load or where the management access isn’t working as expected.

Together, these initial test cases touch on error handling (ICMP unreachable), core protocol operation (LDP), and configuration/state retrieval (MOTD). They are indeed limited in scope, but each maps to a real operational concern: basic IP forwarding and security, MPLS control-plane functionality, and configuration verification.

#### Manual testing

Manual Network Testing is best used for testing and validating network devices for small enterprises. It has some advantages like full control over the router output, where the network administrator can display the required output using show commands. It also provides a human-readable interface in some scenarios and doesn’t require a parsing template to structure the output results as in automated testing. Further, it is easy to use and doesn’t require programming skills.

However, Manual Testing is not scalable in medium and large networks with hundreds or thousands of nodes. It also depends solely on human analysis; therefore, it is subject to errors. Additionally, there is no standard to verify the operability of a protocol or a service, thus, delivery quality will be based entirely on the human factor.

#### Automated testing

Results showed that NETCONF outperformed both classic-CLI and MD-CLI in the test cases, demonstrating faster connection times and test execution due to optimized operations.

NETCONF excels in managing bulk data retrieval and complex configurations, handling large datasets in real-time. The configuration returned using get-config operation on the router used in the test is 65 Kb with more than 1600 lines of data. The get-state output is 3257 Kb with more 69800 lines of data. NETCONF handles and processes this number of lines effectively and in a real-time fashion. Overall, NETCONF’s structured operations significantly enhance network management efficiency over traditional SSH methods.

**Fig. 5:**
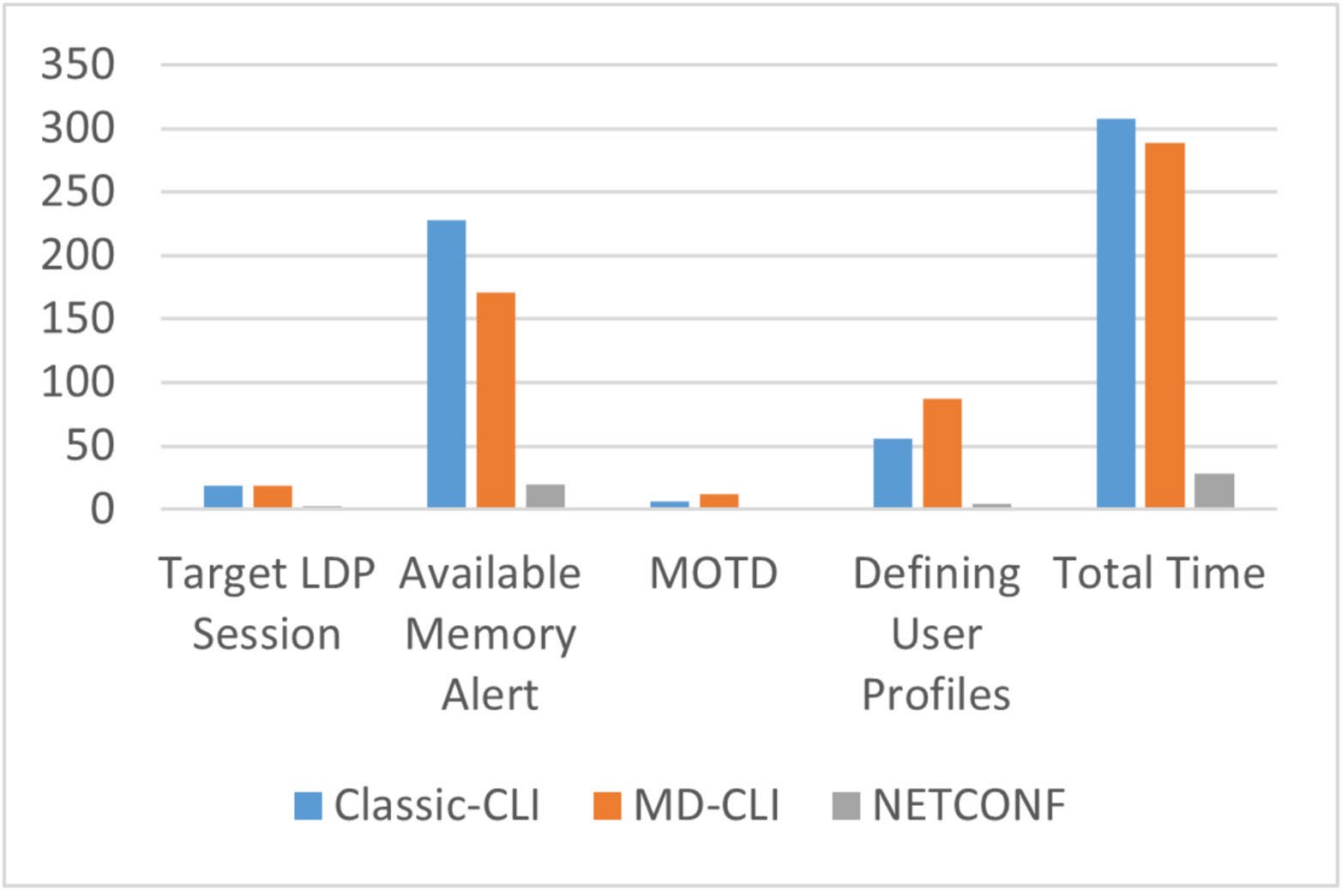
Execution time in seconds for the four test cases in CLASSIC-CLI, MD-CLI, and NETCONF.

In the comparison of test case execution times shown in Fig. 5, NETCONF outperforms MD-CLI by 10.3 times and classic CLI by 11 times. However, developing NETCONF test cases is challenging due to syntax intricacies, context requirements, and higher line counts.

### Discussion

In this section, we address the broader implications of our results and acknowledge certain limitations, especially regarding the use of vendor-specific tools. We also discuss how our findings can generalize to other environments and what trade-offs practitioners should consider when adopting these automation techniques.

One limitation of our study is that some tools we used are proprietary to Nokia and thus not directly applicable to networks with other vendors’ equipment. For instance, Nokia’s Komodo configuration generator and the SROS Automation Testing Suite (SATS) (used for creating Robot Framework test cases) are designed for Nokia SR OS routers. Our entire testbed was built around Nokia’s virtual routers. We want to be transparent that if someone has a Cisco-only or Juniper-only network, they can’t use Komodo or Nokia’s exact testing libraries in that context. We explicitly acknowledge: “Our current implementation relies on Nokia-specific tools, which means the results and workflows demonstrated are tuned to Nokia SR OS devices.”

However, a key point is that our automation approach itself is not fundamentally tied to Nokia—and we aimed to design it in a vendor-agnostic way as much as possible. For configuration generation, our custom Python script uses templates (Jinja2) and an input spreadsheet. This approach can be adapted to any vendor by simply providing different templates. In other words, while we showcased it on Nokia configs, the same script could generate configurations for Cisco IOS or Juniper Junos devices if we supply the corresponding template files. This is actually one motivation for developing our own tool: to avoid vendor lock-in that comes with proprietary solutions. We mention in the paper that by changing the template library, “the Python-based configuration generator can output configurations for various network operating systems, making it a flexible alternative to vendor-specific tools like Komodo.”

For automated testing, we used the Robot Framework with NETCONF and SSH libraries. These are inherently multi-vendor tools. NETCONF, in particular, is an open standard—as long as a device supports NETCONF and has YANG data models, the same Robot Framework tests we wrote (with minor adjustments to device-specific details) could be run against that device. We emphasize this in the discussion: “The NETCONF test cases we developed can be ported to other vendors’ routers (e.g., Cisco, Juniper) with minimal changes, since they rely on standard YANG models and NETCONF operations.” Even our CLI-based tests could be reused on other vendors by altering the command syntax in the Robot scripts or by using vendor-agnostic automation frameworks (for example, Cisco’s pyATS for CLI automation on IOS).

In short, the tools and methods chosen for demonstration were influenced by the equipment we had (Nokia), but the principles and custom code we provide are meant to be vendor-neutral. We have added a note in the Conclusions that we plan to prove this out by extending tests to a multi-vendor testbed (including devices from Cisco or Arista) as part of future work. This will help confirm that our performance findings and the benefits of our Python-based automation hold true beyond a single-vendor environment.

## Conclusions and future work

Introducing automation in telecommunications networks is now essential for enhancing connectivity, operational efficiency, and maintenance practices. Like investing in security measures, investing in automation tools is crucial. This paper explores three key project life cycle steps: topology deployment, configuration generation, and network testing, using various automation methods.

Topology deployment utilized EVE-NG, CLAB, and Pllama emulators. CLAB excelled in flexibility and speed. A YAML generation script for CLAB reduced deployment time from nine minutes to two minutes, enhancing accuracy via Excel-driven inputs.

Moreover, configuration generation integrated Jinja templates with YAML files, leveraging Excel inputs to reduce errors and enhance readability. Komodo tool combined configuration and topology, supporting multi-vendor deployments with error detection capabilities.

Finally, automated testing of network devices outperformed manual methods. CLI, MD-CLI, and NETCONF were compared across 20+ test cases. NETCONF, leveraging YANG models, was 10.3x faster than MD-CLI and 11x faster than CLI, albeit harder to develop due to its structured nature. MD-CLI simplified development with JSON outputs, while CLI offered human-readable outputs.

Overall, this work has proven that using automation in each of the stages of the networks’ deployment can have a huge impact on the time and effort spent in process. Which enables companies to rapidly deploy and increase the quality of life of the users.

Automated reports from test executions provide robust troubleshooting and validation tools. Integrating diverse automation tools throughout a project lifecycle enhances accuracy, quality, operational efficiency, and troubleshooting capabilities. However, there will still be the need for human intervention in cases For future work, the following we list below some of the ideas that can be further investigated:

- Near-term extensions: In the immediate future, we plan to test our automation framework in a multi-vendor environment. This will involve incorporating devices or virtual routers from other vendors (such as Cisco IOS XR or Juniper Junos) into our testbed and verifying that our Python-based configuration generator and automated tests work seamlessly across a heterogeneous network. We will likely need to create new templates or minor adjustments for those platforms, but we expect the core workflow to remain effective. We also intend to integrate our custom automation tools with popular network orchestration platforms like Ansible. For instance, we could use Ansible playbooks to call our Python scripts or orchestrate Containerlab deployments, combining our specialized tools with industry-standard automation pipelines. These near-term steps will help validate and improve the portability and usability of our approach in real-world, diverse networks.
- Long-term research directions: Looking further ahead, one exciting avenue is exploring AI-assisted network automation. We envision using machine learning to optimize certain aspects of automation, such as predicting the best configuration parameters or intelligently analyzing test results to detect anomalies faster. For example, an AI system could learn from past network changes to suggest config tweaks, or automatically identify patterns in telemetry data that indicate performance issues—complementing the rule-based tests we have now. Another long-term goal is to scale up our evaluations to much larger topologies (potentially hundreds of nodes). This might involve leveraging cloud infrastructure to spin up large-scale virtual networks and seeing how tools like EVE-NG, Containerlab, and others perform when the network grows to a significant size. Scaling tests will help uncover any performance bottlenecks or management challenges that aren’t apparent in a 6-node scenario. Additionally, we are interested in exploring streaming telemetry and model-driven monitoring (e.g., gNMI/gRPC-based telemetry) as part of the automation loop—integrating real-time network state information with the kind of automated testing we performed, to create a more continuous and self-correcting network management system.

## Data Availability

No datasets were generated or analysed during the current study.
